# SLEEP QUALITY AS A MECHANISM LINKING PHYSICAL ACTIVITY AND COGNITIVE FUNCTIONING

**DOI:** 10.1093/geroni/igz038.2866

**Published:** 2019-11-08

**Authors:** Alycia N Bisson, Margie E Lachman

**Affiliations:** Brandeis University, Waltham, Massachusetts, United States

## Abstract

Modifiable health behaviors, such as physical activity and sleep quality are important for cognition throughout life. A growing body of research also suggests that engaging in enough physical activity is important to sleeping well. One recent study found that sleep efficiency mediates the relationship between physical activity and cognition. It is still unknown whether other metrics of sleep quality are mediators. The present study tested mediation in the second wave of the Midlife in the United States (MIDUS) study. Using the PROCESS macro for SPSS, we found that those who were more physically active fell asleep faster, and had better executive functioning. In addition, those who were more physically active reported waking up fewer times during the night, and had better executive functioning and self-rated memory. Discussion will focus on the moderating role of gender and distinctions between findings with different measures of sleep, physical activity, and cognition.

